# Physiological targets of salicylic acid on *Artemisia aucheri* BOISS as a medicinal and aromatic plant grown under in vitro drought stress

**DOI:** 10.1186/s40529-016-0154-6

**Published:** 2016-12-08

**Authors:** Jalil Abbaspour, Ali Akbar Ehsanpour

**Affiliations:** Department of Biology, Faculty of Science, University of Isfahan, Isfahan, Iran

**Keywords:** *Artemisia aucheri*, Drought stress, Salicylic acid, Polyethylene glycol, Antioxidant enzyme

## Abstract

**Background:**

*Artemisia aucheri* BOISS is a medicinal and aromatic plant, which is endemic to mountainous areas of Iran and surroundings. In this study, we investigated the alleviating effects of salicylic acid (SA) pretreatment (0.01 and 0.1 mM) on *A. aucheri* under in vitro drought stress induced by 2 and 4% polyethylene glycol (PEG/6000).

**Results:**

Plants exposed to PEG stress showed higher levels of H_2_O_2_, MDA and electrolyte leakage compared with control. While SA pretreatment decreased these parameters under PEG stress significantly. The activity of CAT, POD, APX, SOD and GR positively changed with PEG and more induction in activity of antioxidant enzymes was observed in SA-pretreated plants under PEG stress. Furthermore, ASA, GSH and their redox ratios (ASC/DHA and GSH/GSSG) enhanced with SA pretreatments. Analysis of our data revealed that MDA, DHA and H_2_O_2_ were the best targets for SA under in vitro PEG treatment for *A. aucheri* plants.

**Conclusions:**

Salicylic acid as a signal molecule mitigated adverse effects of PEG-simulated drought stress on *A. aucheri* under in vitro condition by improving the activity of antioxidant enzymes. In addition, protective role of SA was also related to promotion of ascorbate–glutathione cycle.

## Background

*Artemisia aucheri* BOISS is a medicinal and aromatic plant belongs to Asteraceae family. There are around 500 species of Artemisia in Asia, Europe and North America and 34 species of this family are found all over Iran (Mozaffarian 2010)*. Artemisia aucheri* is limited mostly to mountainous landscapes with high slope, sandy soils and with annual precipitation of 300–450 mm (Hosseini et al. 2013). Morphological features of this plant consist of perennial, suffrutescent, stem numerous and erect, indumentum white-tomentose, leaves pinnate or bipinnate, capitula arranged in a panicle-congested, calathium sessile and ovate, phyllaries imbricate and multiseriate, florets 3–4 (Podlech 1986). This plant has many medicinal properties and is useful in traditional medicines for the treatment of some diseases. Some findings indicate that *A. aucheri* extraction have cytotoxic effects in cancer treatment (Ghazi-Khansaria et al. 2013). In addition, anti leishmanial effects of this plant have been studied (Sharif et al. 2006). Verbenone, camphor, 1,8-cineole, trans-verbenol, chrysanthenone, mesitylene, α-pinene, acyclic monoterpenes, and monoterpenehydroperoxides are bioactive compounds that can be extracted from this plant (Hashemi et al. 2007; Rustaiyan et al. 1987).

Water stress is one of the major environmental stresses that limit plant growth and development around the world. Plants have advanced complex mechanisms at cellular and molecular levels to mitigate negative effects of water deficiency (Chaves et al. 2009; Shen et al. 2014). Drought stress induces generation of reactive oxygen species (ROS) leading to oxidative stress (Bartels and Sunkar 2005). When ROS accumulate in plant tissues, it damages lipids, proteins, DNA and consequently leading to cell death (Molassiotis et al. 2006). To alleviate the deleterious effects of ROS, plants developed an antioxidant defense system including both enzymatic such as superoxide dismutase (SOD), ascorbate peroxidase (APX), catalase (CAT), peroxidase (POD), glutathione reductase (GR), and non-enzymatic antioxidants including ascorbate and glutathione (Foyer and Noctor 2005; Gill and Tuteja 2010; Mittler 2002; Suzuki et al. 2012). It seems that the balance between ROS production and capability of scavenging ROS by antioxidant system affects on drought tolerance of plant (Boaretto et al. 2014).

Salicylic acid (SA) is a natural phenolic compound and plant growth regulator that plays a key role in the regulation of plant growth and development (Rivas-San Vicente and Plasencia 2011). Previous studies have shown some roles of SA in biotic stresses via induction of systemic acquired resistance. In addition to its role in biotic stress responses, SA also participates in modulating the plant response to many abiotic stresses, including salinity (Rady and Mohamed 2015), cold (Luo et al. 2014), drought (Ying et al. 2013) and the excess of heavy metals (Shakirova et al. 2016). SA as a signal molecule increases the activity of enzymatic antioxidant and reduce the production of reactive oxygen species (ROS) (Horváth et al. 2007). It has been found that exogenously application of SA enhances both enzymatic and non-enzymatic systems under water stress and consequently improves drought resistance. Furthermore, exogenous application of SA decreased the damaging effect of drought on plants by maintaining the integrity of the plasma membrane, which evaluate by reduction of malondialdehyde (MDA) and electrolyte leakage (Kadioglu et al. 2011). However, most of the reports have focused on the roles of SA in field condition and there is little information about its role in modification of antioxidant system under in vitro condition. On the other hand, tissue culture technology is a rapid and fast method in assessment of physiological responses of plant to phytohormones, elicitors and abiotic stresses (Ramakrishna and Ravishankar 2011).

Polyethylene glycol (PEG) has been widely used to mimic drought in many studies to investigate plant adaptive mechanisms (Hajihashemi et al. 2013; He et al. 2014). Despite the relatively great number of reports on the compounds and medicinal properties of *A. aucheri*, there is no report on the physiological and biochemical responses of this plant to PEG as well as possible positive effects of SA treatment. Therefore, in the present study PEG-6000 and SA were used (1) to understand the effect of oxidative stress induced by water stress on *A. aucheri* (2) to determine which physiological parameter/s is more responsive to PEG and SA as the best target.

## Methods

### Plant materials and treatments

The *A. aucheri* BOISS plants were obtained from Department of Biology, University of Isfahan, Isfahan, Iran. For multiplication, plants were grown on MS medium (Murashige and Skoog 1962) supplemented with 30 g l^−1^ sucrose and 8 g l^−1^ agar (pH 5.8) then all cultures were kept at 25 ± 1 °C with 16/8 h photoperiod under approx. 44 µmol phot m^−2^ s^−1^ light.

As pretreatment, in vitro grown explants were cultured on MS medium containing, 0, 0.01 and 0.1 mM SA for 1 week, plants were then transferred to MS medium supplemented with 0, 2 and 4% (w/v) PEG (MW 6000) for 2 weeks. PEG was added to MS medium according to diffusion based method described by Girma and Krieg (1992). The water potentials of media (MS medium supplemented with and without PEG) were: −0.3 MPa for control; −0.45 MPa for 2% PEG and −0.6 MPa for 4% PEG.

### H_2_O_2_ content

Hydrogen peroxide content was determined according to Velikova et al. (2000). Fresh leaves (500 mg) were homogenized with 5 ml of 0.1% (w/v) trichloroacetic acid (TCA). The homogenate was centrifuged at 12,000*g* for 15 min. Then, 0.5 ml of the supernatant was added to 0.5 ml 10 mM potassium phosphate buffer (pH 7.0) and 1 ml of 1 M KI. After incubation of samples for 15 min at room temperature, the absorbance of samples was recorded at 390 nm. The H_2_O_2_ contents were calculated using a standard curve and results were expressed as μmol g^−1^ FW.

### Lipid peroxidation

Lipid peroxidation was measured by method of Heath and Packer (1968). Leaf samples (0.2 g) were homogenized in 5 ml of trichloroacetic acid (TCA, 0.1% w/v) and centrifuged at 10,000*g* for 15 min at 4 °C. To each 1 ml aliquot of the supernatant, 4 ml of 20% TCA containing 0.5% thiobarbituric acid (TBA) was added. The mixture was incubated for 30 min at 95 °C and then quickly cooled in an ice bath. The absorbance of the samples was recorded at 532 and 600 nm. The malondialdehyde (MDA) content was calculated using an extinction coefficient of 155 mM^−1^ cm^−1^ and the results expressed as nmol g^−1^ FW.

### Electrolyte leakage determination

The membrane injury under PEG treatment was estimated by electrolyte leakage as described by Lutts et al. (1995) method. Leave samples (0.2 g) were placed in test tubes containing 10 ml of double distilled water. Leaf discs were incubated on shaker at 25 °C for 6 h then electrical conductivity (EC_1_) was measured. Samples were then autoclaved at 120 °C for 20 min and after cooling the solution at room temperature, the final electrical conductivity (EC_2_) was recorded. The electrolyte leakage (EL) was calculated by using the following formula:

$$ {\text{EL}} = \left( {{\text{EC}}_1/{\text{EC}}}_2 \right) \times 100 $$

### Antioxidative enzyme activities

To extract antioxidative enzymes, fresh leaf samples (1 g) were homogenized using a chilled mortar and pestle in 5 ml of Na-phosphate buffer (100 mM, pH 7.8) containing EDTA (1 mM), dithiothreitol, (DTT, 1 mM) and polyvinylpyrrolidone (PVP, 2% w/v). The homogenates were centrifuged at 13,000*g* for 20 min at 4 °C. The supernatant was used for the assays of antioxidative enzyme activities using UV–visible spectrophotometer (Shimadzu, Japan). Protein content was determined according to Bradford (1976), using bovine serum albumin (BSA) as standard.

Catalase activity (CAT, EC 1.11.1.6) was assayed according to the method of Aebi (1984). The reaction mixture in a total volume of 1 ml consisted of 0.95 ml of 50 mM sodium phosphate buffer (pH 7.0), 10 mM H_2_O_2_ and 50 μl of enzyme extract. The decrease in the absorbance of H_2_O_2_ was recorded at 240 nm for 1 min. The enzyme activity was calculated using an extinction coefficient of 39.4 mM^−1^ cm^−1^ and expressed as µmol decomposition of H_2_O_2_ min^−1^ mg^−1^ protein.

Peroxidase (EC 1.11.1.7) activity was measured by method of Plewa et al. (1991). The assay mixture was consisted of 1.99 ml 50 mM sodium phosphate buffer (pH 7.0) supplemented with 0.1 μM EDTA, 10 mM guaiacol and 15 mM H_2_O_2_ and 100 μl of the enzyme extract in a total volume of 2 ml. Guaiacol oxidation and production of tetraguaiacol was monitored by increase in absorbance of 470 nm. The activity of POD was calculated by extinction coefficient of tetraguaiacol (26.6 mM^−1^cm^−1^) and expressed as µmol tetraguaiacol min^−1^mg^−1^protein.

Activity of APX (EC 1.11.1.11) was determined by recording the decrease in absorbance of ascorbate at 290 nm as described by Nakano and Asada (1986). The reaction mixture (1.0 ml) contained 0.95 ml of 50 mM sodium phosphate buffer (pH 7.0), 0.5 mM ascorbic acid, 0.2 mM EDTA, 0.2 mM H_2_O_2_ and 50 μl of enzyme extract. APX activity was calculated by using the extinction coefficient 2.8 mM^−1^ cm^−1^ and expressed as µmol decomposition of ascorbate min^−1^ mg^−1^ protein.

Superoxide dismutase (SOD, EC 1.15.1.1) activity was measured by monitoring the inhibition of the photochemical reduction of nitro blue tetrazolium (NBT) as described by Beauchamp and Fridovich (1971). The reaction mixture, consisted of 50 mM Na-phosphate buffer (pH 7.8), 0.1 mM EDTA, 13 mM methionine (Sigma-Aldrich Corporation), 75 μM NBT (Sigma-Aldrich Corporation), 2 μM riboflavin (Sigma-Aldrich Corporation), and 50 µl of enzyme extract in a test tubes, were incubated for 15 min under fluorescent. One unit of SOD activity was defined as the amount of enzyme that inhibited 50% of NBT photochemical reduction and expressed as unit mg^−1^ protein.

Activity of GR (EC 1.6.4.2) was determined based on the method of Smith et al. (1988). The reduction of 5,5′-dithiobis-2-nitrobenzoic acid (DTNB) to TNB was measured by reduced glutathione (GSH). The reaction mixture (1.0 ml) consisted of 0.980 ml of 50 mM potassium phosphate buffer (pH 7.5), 0.5 mM EDTA, 0.75 mM DTNB (Sigma-Aldrich Corporation), 0.1 mM NADPH (Sigma-Aldrich Corporation), 1 mM oxidized glutathione (GSSG) (Sigma-Aldrich Corporation) and 0.01 ml of the enzyme extract. The rate of increase in the absorbance of 412 nm was recorded for 1 min. The enzyme activity was calculated using an extinction coefficient of 6.2 mM^−1^ cm^−1^ and expressed as µmol of TNB min^−1^ mg^−1^ protein.

### Ascorbate and glutathione contents

Estimation of the ascorbate (ASC), dehydroascorbate (DHA) and total ascorbate was performed by method of Law et al. (1983). Fresh leaf samples (0.2 g) were homogenized in 2 ml of 6% (w/v) trichloroacetic acid (TCA) using a chilled mortar and pestle and centrifuged at 15,000*g* for 15 min at 4 °C. To estimate the total ascorbate, 200 μl of 150 mM Na-phosphate buffer (pH 7.4) and 200 μl of 10 mM DTT were added to 200 μl of the supernatant. The mixture was incubated at room temperature for 10 min, then mixed with 100 µl of 0.5% *N*-ethylmaleimide (NEM). ASC content was determined in the same way, except that DTT and NEM were replaced with water. To each of samples were then added 400 µl of 10% (w/v) TCA, 400 µl of 44% (v/v) H_3_PO_4_, 400 µl of 4% (w/v) α-α′-bipyridyl dissolved in 70% ethanol and 200 ml of 3% FeCl_3_. After shaking, samples were incubated at 37 °C for 60 min and absorbance was recorded at 525 nm on a UV–Vis Spectrophotometer (Shimadzu, Japan). The difference between the total ascorbate and ASC content was used to calculate DHA content. The concentrations of total ascorbate and ASC were calculated using a standard curve with known amount of ASC and expressed in μmol g^−1^ FW.

To extract glutathione, fresh leaves (0.2 g) were homogenized in 2 ml of 5% (w/v) sulphosalicylic acid using a chilled mortar and pestle. The homogenate was centrifuged at 10,000*g* for 10 min at 4 °C and supernatant was used for estimation of reduced (GSH), oxidised (GSSG) and total glutathione according to Anderson (1985) method. Total glutathione (GSH + GSSG) was measured using an assay mixture containing 143 mM sodium phosphate buffer (pH 7.5), 6.3 mM EDTA, 0.3 mM NADPH and 150 µl of the supernatant, in total volume 0.85 ml. To estimate GSSG content, before adding this buffer, 10 μl of 2-vinylpyridine and 20 µl of triethanolamine mixed with 150 supernatant. Then, 100 µl of DTNB (6 mM) and five unit of glutathione reductase were added and the absorbance of samples were recorded at 412 after 5 min. Total glutathione and GSSG were determined using a standard curve prepared using GSH. The amount of GSH was calculated as the difference between total glutathione and GSSG.

### Statistical analysis

All experiments were performed with at least three independent replicates. Data were analyzed for significance of differences between means with Duncan’s test at P < 0.05 (Two-way ANOVA). SPSS software (version 16) was used for Statistical analysis of data and results were expressed as mean ± standard deviation (SD). The standardization of values was also carried out by SPSS software using the following formula.

$$ z_{i} = \frac{{X_{i} - \bar{X}}}{S} $$

X_i_ = each data point i, X = the average of all the sample data points, S = the sample standard deviation of all sample data points, z_i_ = the data point i standardized to s, also known as Z-score.

## Results

### H_2_O_2_ content, lipid peroxidation and electrolyte leakage

**Fig. 1:**
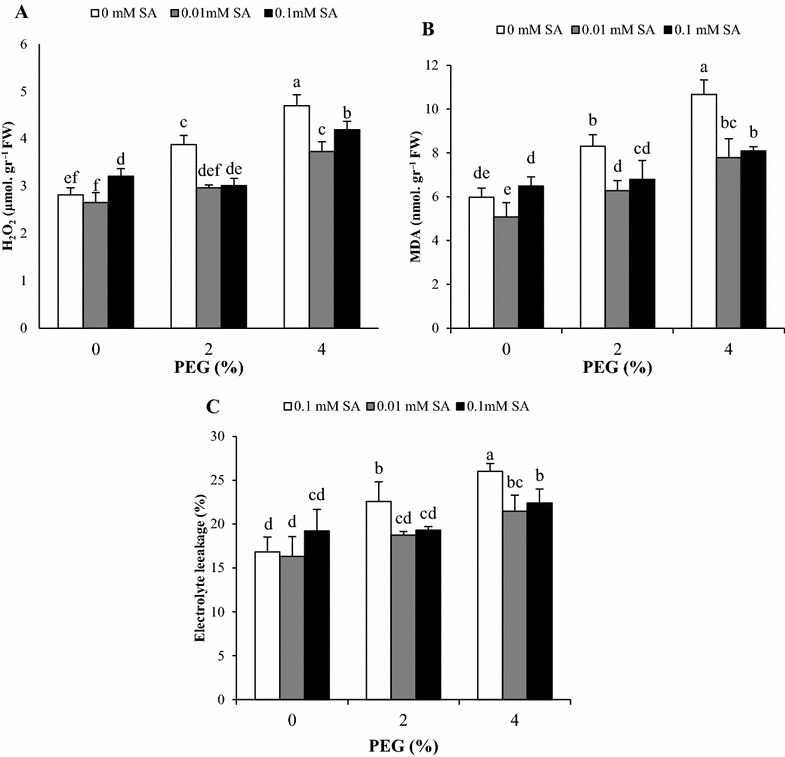
Effects of SA pretreatment and PEG on **A** H_2_O_2_, **B** MDA content and **C** electrolyte leakage of *Artemisia aucheri*. Data are mean ± SD of three replicates (n = 3). *Different letters* indicate significant (P < 0.05) based on Duncan’s test

H_2_O_2_ level of pretreated plant with 0.1 mM SA was significantly higher than control plants, without PEG stress. Increasing of PEG concentration induced a dramatic increase in H_2_O_2_ production. SA pretreatments had a positive effect in reduction of H_2_O_2_ content under 2 and 4% PEG compared with plants without SA pretreatment (Fig. 1A). Malondialdehyde (MDA) content and electrolyte leakage was measured as an index of injury to plasma membrane under stress condition. Data showed that MDA content significantly enhanced in PEG-treated plants as highest increase was observed in 4% PEG. In plants pretreated with SA (0.01 and 0.1 mM), MDA content significantly decreased under the drought Stress (Fig. 1B). Electrolyte leakage had the similar trend with MDA content as it significantly elevated by PEG and decreased in SA pretreated plants. SA pretreatment had no significant effect on MDA content and electrolyte leakage of control plant without PEG stress (Fig. 1B, C).

### Antioxidative enzyme activities

**Fig. 2:**
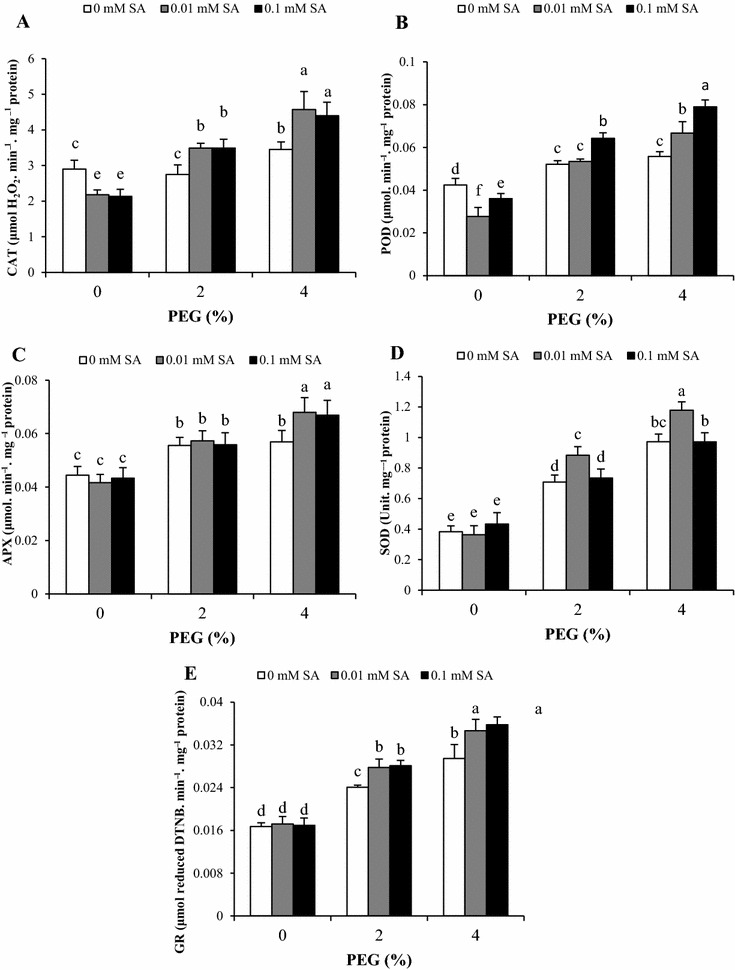
Effects of SA pretreatment and PEG on activities of **A** CAT, **B** POD, **C** APX, **D** SOD and **E** GR of *Artemisia aucheri*. Data are mean ± SD of three replicates (n = 3). *Different letters* indicate significant (P < 0.05) based on Duncan’s test

Increase of antioxidant enzymes such as CAT, APX, POD, SOD and GR is one of the most common defense responses of plants against oxidative. As shown in Fig. 2, SA pretreatment induced activity of all antioxidant enzymes under PEG stress. In the presence of SA, CAT activity significantly declined in control plant without PEG treatment. *Artemisia aucheri* plants showed more CAT activity at 4% PEG but not at 2% compared with control plants. Pretreatments of SA (0.01 and 0.1 mM) induced the higher CAT activities than PEG treatments without SA (Fig. 2A). Water stress resulting from PEG significantly increased activity of APX in both levels of PEG. SA pretreatment had no effect on in control and plant treated with 2% PEG, but SA significantly improved APX activity under high level of PEG (Fig. 2C). Similar to CAT, pretreatments with 0.01 and 0.1 mM SA significantly reduced POD activity in control plant without PEG treatment and lower activity was observed at 0.01 mM SA. Plants subjected to 2 and 4% PEG demonstrated similar increase in activity of POD compared with the control. Under 2% PEG, the plant pretreated with 0.1 mM SA showed higher POD activity, while 0.01 mM SA had no significant effect. In the presence of 4% PEG, the POD activity noticeably enhanced with SA pretreatments and culminated at 0.1 mM SA (Fig. 2B). The exposure of *A. aucheri* to PEG significantly increased SOD almost based on a PEG concentration dependent manner. The effectiveness of SA pretreatment in increasing SOD activity was only observed at 0.01 mM SA. Moreover, activity of SOD in untreated as well as pretreated plants was not different in the absence of PEG (Fig. 2D). Plant grown with 2 and 4% PEG demonstrated higher levels of GR activity compared with control plants. SA pretreatment plus PEG increased GR activity more than PEG without SA and the effect of both concentrations of SA was almost the same. However, SA pretreatment had no significant effect on GR activity under normal condition (Fig. 2E). In the present study, it is interesting that, positive effects of SA on some antioxidant enzymes under the same PEG levels were similar and not depending on the SA concentration.

### Ascorbate and glutathione contents

**Fig. 3:**
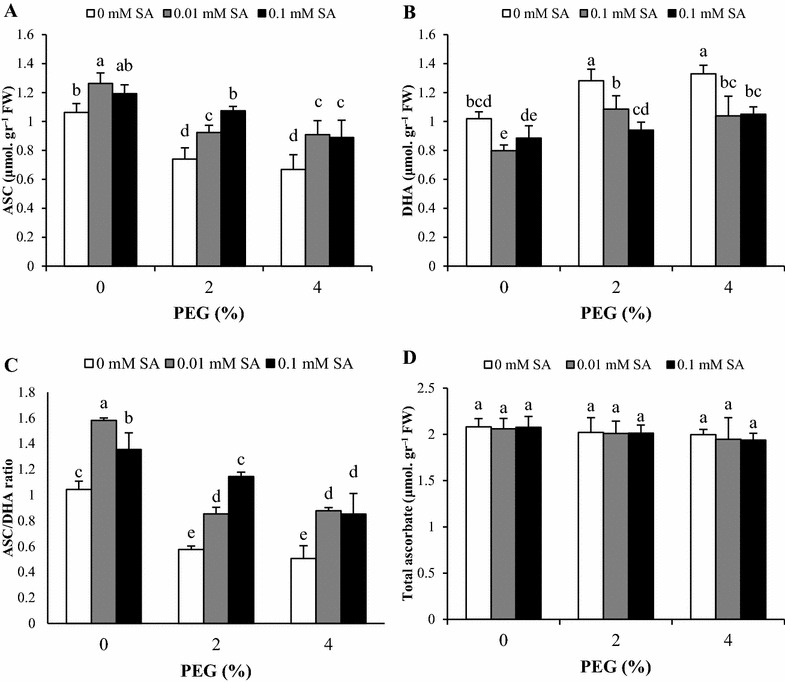
Effects of SA pretreatment and PEG on **A** ASC, **B** DHA, **C** ASC/DHA ratio and **D** total ascorbate of *Artemisia aucheri*. Data are mean ± SD of three replicates (n = 3). *Different letters* indicate significant (P < 0.05) based on Duncan’s test

**Fig. 4:**
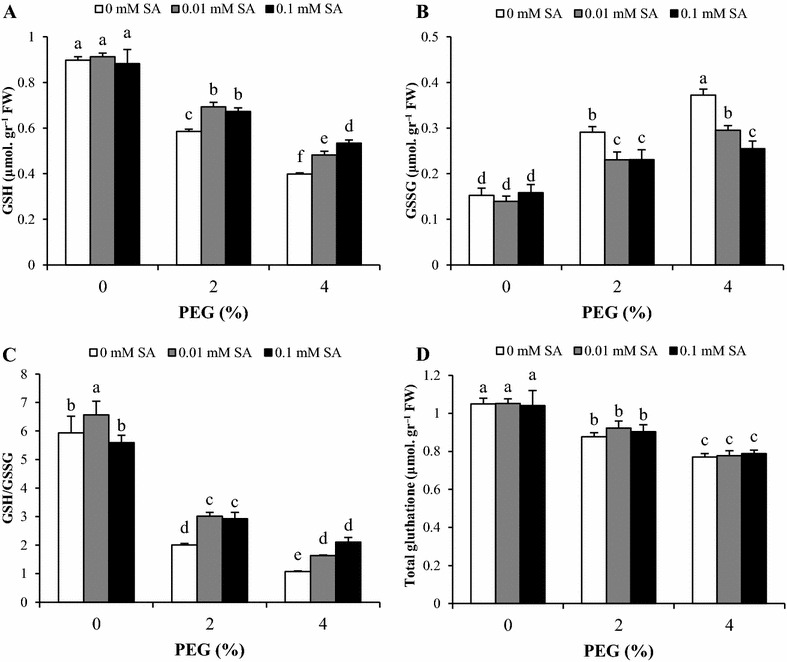
Effects of SA pretreatment and PEG on **A** GSH, **B** GSSG, **C** GSH/GSSG ratio and **D** total glutathione of *Artemisia aucheri*. Data are mean ± SD of three replicates (n = 3). *Different letters* indicate significant (P < 0.05) based on Duncan’s test

The effects of drought and SA pretreatments on ascorbate and glutathione pools of *A. aucheri* are illustrated in Figs. 3 and 4. In the absence of PEG, SA pretreatment at 0.01 mM increase ASC and ASC/DHA ratio significantly. A similar pattern was obtained in ASC content and ASC/DHA ratio under both concentrations of PEG. By contrast, DHA content showed similar increase in 2 and 4% PEG. In comparison with 2% PEG, SA pretreatments plus 2% PEG significantly enhanced ASC content and ASC/DHA in dose dependent manner but decreased DHA content. In addition, plant subjected to 4% PEG and SA pretreatments (0.01 and 0.1 mM) showed higher ASC content and ASC/DHA ratio against 4% PEG without SA pretreatments, but there was no difference at 0.01 and 0.1 SA. The values of total ascorbate weren’t affected by PEG-simulated drought stress and SA pretreatment.

Under non-stress condition, glutathione pools did not change with SA pretreatments, except for GSH/GSSG which increased with 0.01 mM SA. GSH content and GSH/GSSG ratio decreased gradually with increasing of PEG concentrations. Combination of SA pretreatments at 0.01 and 0.1 mM in the medium with 2% PEG increased GSH content and GSH/GSSG ratio equally compared to 2% PEG without SA pretreatments. In contrast, a similar decrease was observed in GSSG content by 2% PEG plus 0.01 and 0.1 mM SA compared with 2% PEG without SA pretreatments. In plants treated with 4% PEG, SA pretreatment caused a decrease in GSSG content in a dose-dependent manner. The opposite trend was found in GSH content and GSH/GSSG ratio. Total glutathione reduced under PEG stress and maximum reduction was observed at high level of PEG (4%), but SA had no alleviating effect on total glutathione (Fig. 4).

### Comparing of biochemical indicators based on standardization of their values

To identify the best biochemical targets for SA and PEG treatment in *A. aucheri* plants, we standardized the data obtained from different biochemical measurements (Table 1) based on the Z-score of the variables. According to Table 1, the comparison of standard values indicated SA at 0.01 and 0.1 mM (in control without PEG treatments) positively affected ASC/DHA ratio more than other parameters. PEG stress at 2 and 4% alone showed maximum negative effect on GSH/GSSG and GSSG, respectively. The interaction of 0.01 mM SA with 2% PEG had a maximum effect in decreasing of H_2_O_2_ compared to the other indicators, while interaction of 0.1 mM SA with both concentrations of PEG was more effective on decreasing of DHA. Furthermore, the reduction of MDA content under 4% PEG with 0.1 mM SA was more noticeable compared to other biochemical indicators (Table 1).

**Table 1:**
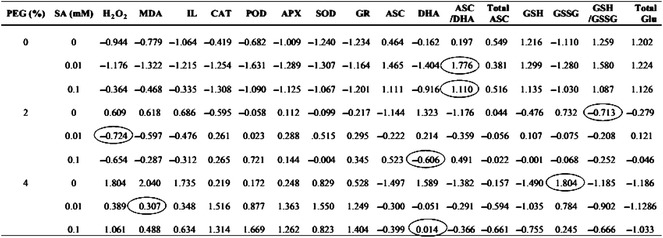
Biochemical targets of SA pretreatment and PEG on *Artemisia aucheri* by comparing of standard values in each row (marked by circle) based on Z-score of the variables

Circle indicates the maximum difference with the same level of PEG treatment. Positive values indicate data above, and negative values shows data below the average (zero). Data are mean of three replicates

## Discussion

Under drought stress, plant display some physiological and biochemical responses to cope with oxidative damage. These responses include enzymatic and non-enzymatic defense systems leading to stress tolerance. Increasing evidences show that phytohormones play an important role in drought tolerance. Salicylic acid is one of the important plant growth regulators which has a critical role in modulating responses to drought (Kang et al. 2014; Miura and Tada 2014). To understand the possible positive effects of SA pretreatment during PEG stress, we investigated this plant hormone on *A. aucheri* under PEG treatment in in vitro condition.

Our results indicated PEG-simulated drought stress increased H_2_O_2_ generation in *A. aucheri* plant. Similarly, SA pretreatment at 0.1 mM concentration caused H_2_O_2_ accumulation in normal condition, while SA at both concentrations reduced the production of H_2_O_2_ in PEG treated *A. aucheri* plants. According to previous studies, increasing of H_2_O_2_ content was predictable because PEG-simulated drought stress induces oxidative stress by generation of reactive oxygen species (Hajihashemi et al. 2013; Li et al. 2011). Moreover, reduction in the H_2_O_2_ accumulation by application of salicylic acid under water stress has been previously reported in other plants (Nazar et al. 2015). By contrast, it has been proved SA as a signal molecule accumulated low levels of H_2_O_2_ (Harfouche et al. 2008). Therefore, it seemed that the increase in H_2_O_2_ content with 0.1 mM SA pretreatment in non-stress condition (without PEG treatment) associated with dual role of this signal molecule to induction of drought resistance (Miura and Tada 2014). In addition, SA at 0.01 mM was more effective than 0.1 mM in H_2_O_2_ reduction under high level of PEG show in that the effectiveness of low concentration in vitro condition. Electrolyte leakage and malondialdehyde (MDA) content are indicators that reflect the degree of membrane injury. In our study, increase of PEG stress enhanced these parameters in parallel with H_2_O_2_ content, whereas SA pretreatments successfully ameliorated these negative effects. Membrane damage in stress condition is correlated with high H_2_O_2_ levels, which increase lipid peroxidation and membrane permeability (Mittler 2002). The decrease in electrolyte leakage and MDA content by SA pretreatment can be related to the promotion of antioxidant defense system and also to the lower levels of H_2_O_2_ in presence of SA under PEG stress. Our findings are consistent with those reported by other authors (Feng et al. 2003; Ying et al. 2013).

Antioxidative enzyme activities plays vital roles in drought tolerance by reduction of ROS. Exposure of *A. aucheri* plant to PEG led to increase with some variation in antioxidative enzyme activity including CAT, APX, POD, SOD and GR. The present results are also supported by previous observations in several plant species such as cucumber (Li et al. 2011), alfalfa (Wang et al. 2009), Kentucky bluegrass (Bian and Jiang 2009) and cotton (Sekmen et al. 2014). SOD catalyzes the conversion of superoxide radicals to O_2_ and H_2_O_2_, while CAT, POD and APX catalyze dismutation reactions of H_2_O_2_ into H_2_O (Mittler 2002). In this study, SA pretreatments positively changed antioxidative enzyme activities compared to non SA-pretreated plants under PEG stress. In case of CAT, APX and GR, similar increase was obtained at two SA concentrations, while it was different to the other enzymes. It seemed that SA had no effect on APX activity under low level of PEG (2%) but it was effective in 4% PEG. This result may indicate that the increase in APX activity without SA have been sufficient to cope with oxidative stress under low PEG stress, while SA has improved CAT activity in this condition due to low activity. Under PEG stress, SA at 0.01 mM concentration stimulated SOD activity while POD enzyme was induced by 0.1 mM SA pretreatment. It probably reflects different influences on SOD and POD activity under in vitro condition. Generally, Positive effects of SA pretreatment on antioxidant enzymes in this study is in accordance with the findings obtained in sensitive and tolerant maize cultivars to drought (Saruhan et al. 2012). Furthermore, it has been previously reported that SA alleviates drought stress by induction of antioxidant enzymes such as CAT, APX, SOD, POD and GR (Habibi 2012; Kadioglu et al. 2011). It seemed that the increase of antioxidative enzyme activities by SA pretreatment, as a potential mechanism against water deficiency, promoted the ability of *A. aucheri* in ROS scavenging and resulting in a better resistance to PEG stress. Notably, our results also revealed that SA pretreatments inhibited CAT and POD activity in non-stressed condition. It has been proved that SA prevented CAT and POD activities and increased ROS accumulation (Horváth et al. 2002; Khokon et al. 2011). On the other hand, SA pretreatment with temporary inhibition of CAT and POD led to increase of H_2_O_2_ level in *A. aucheri*. H_2_O_2_, as a signal molecule, induced an adapting mechanism by activation of antioxidative enzymes in PEG condition leading to stress resistance. Additionally, our data indicated that plant exposed to 2% PEG without SA exhibited higher APX activity without significant change in activity of CAT which could be explained by differences in affinity for H_2_O_2_ as substrate of CAT and APX. On the other hand, the H_2_O_2_ content in plants grown in 2% PEG only is sufficient to increase APX activity because this enzyme has high affinity with H_2_O_2_. Unlike APX, CAT has low affinity with H_2_O_2_, thereby only removing the high concentration of H_2_O_2_ (Willekens et al. 1997). Noticeably, both concentrations of SA had similar effects on some antioxidant enzymes as targets under the same levels of in vitro PEG treatment.

The ascorbate–glutathione cycle is one of essential mechanism to eliminate ROS in plant exposed to stress (Drążkiewicz et al. 2003). APX and GR are two key enzymes involved in this pathway. The reduction of oxidized glutathione (GSSG) to reduce form (GSH) is mediated by the activity of GR (Noctor and Foyer 1998). Under PEG stress, our data revealed a decrease in reduced forms of ascorbate (ASC) and glutathione (GSH), resulting in a decrease in ASC/DHA and GSH/GSSG ratio, particularly in plants grown at 4% PEG compared to control plants. The opposite trend was observed in DHA and GSSG content. These changes could be the result of ROS accumulation caused by PEG stress. Furthermore, elevated APX activity under PEG stress needs ASC as electron donor to produce DHA. It can be speculated that, based on the ascorbate–glutathione cycle, in addition to APX activity, the activity of GR and DHAR is effective on ASA and DHA and other components of this cycle. ASA as a substrate for APX activity is product of dehydroascorbatereductase (DHAR), where DHA is converted to ASA. Consequently, the changes of ASA and DHA under PEG stress and SA pretreatment might be related to DHAR activity while, APX activity is more correlated to changes of H_2_O_2_ content. Diminished GSH content with PEG may be the result of DHA reduction by enhanced dehydroascorbatereductase (DHAR) activity under oxidative stress and increasing of GR activity could not alleviate this reduction. In addition to, it proves with the decreased levels of total glutathione in present of PEG. Similar to our observations, it has been found that PEG reduces ASC, GSH and their redox ratios (ASC/DHA and GSH/GSSG) in tolerant and sensitive rice seedlings (Pyngrope et al. 2013). Pretreatment of *A. aucheri* with SA resulted in enhanced levels of ASC and GSH under PEG stress condition. SA also raised ASC/DHA and GSH/GSSG ratio, while DHA and GSSG contents were reduced. Furthermore, our data showed the beneficial effect of 0.1 mM SA pretreatment compared with 0.01 mM. Similar results were obtained by Kadioglu et al. (2011) in *Ctenanthe setosa* GSH and ASC as a low molecular weight antioxidants contribute in activation of various defense and involve in scavenger of ROS (Gill and Tuteja 2010; Szalai et al. 2009). Moreover, it is known that maintaining of sustained level of GSH is necessary to cope with oxidative stress (Pyngrope et al. 2013). These findings suggested that SA with promotion of GR activity increased GSH content under stress condition due to conversion of GSSG to GSH (Kadioglu et al. 2011; Nazar et al. 2011). Since ASC is a substrate for APX activity, SA pretreatment raised the ASC/DHA ratio and modulated antioxidant capacity for ROS scavenging to meet the challenge of oxidative stress under drought. Our data suggest that SA improves the tolerance of *A. aucheri* to PEG-simulated drought Stress by enzymes and metabolites of the ascorbate–glutathione cycle. Our results demonstrated that, the total ascorbate was stable under PEG stress and SA treatment. In fact, PEG and SA changed components of ascorbate pool (ASA and DHA) not the total ascorbate, resulted in change of redox ratios (ASC/DHA and GSH/GSSG). In contrast, total glutathione progressively reduced with increase of PEG concentration, which showed destructive effects of PEG on glutathione biosynthesis.

The comparison of measured biochemical indicators in this study showed that the change of ASC/DHA ratio is a suitable indicator to assess the positive effect of SA on untreated plants with PEG. According to our results, the increase of GSH/GSSG ratio and GSSG content in *A. aucheri* plant treated with PEG were two suitable indicators to determine adverse effect of PEG. Since SA, strongly reduced H_2_O_2_, DHA and MDA content, we suggest evaluation of these biochemical parameters are the best indicators for drought stress tolerance of *A. aucheri* after SA treatment.

## Conclusions

In summary, exposure of *A. aucheri* plant to drought stress induced H_2_O_2_, MDA, electrolyte leakage and elevated the activity of CAT, POD APX, SOD and GR. SA pretreatment at 0.1 and 0.01 mM SA before the onset of PEG stress efficiently decreased H_2_O_2_, MDA and electrolyte leakage. Alleviating effects of SA pretreatments in *A. aucheri* plant exposed to PEG could be associated with the promotion of antioxidative enzyme activities and ascorbate–glutathione cycle resulted in tolerance to PEG stress. In addition, the changes of H_2_O_2_, DHA and MDA content reflected positive effect of SA pretreatment in PEG stress condition rather than other indicators.

## Abbreviations

ROS: reactive oxygen species
H_2_O_2_: hydrogen peroxide
MDA: malondialdehyde
CAT: catalase
POD: peroxidase
APX: ascorbate peroxidase
SOD: superoxide dismutase
GR: glutathione reductase
ASA: ascorbate
DHA: dihydroxyascorbate
GSH: reduced glutathione
GSSG: oxidized glutathione
